# Enhancing Growth and Gut Health in Squabs: The Impact of Fermented Mixed Feed

**DOI:** 10.3390/ani14101411

**Published:** 2024-05-08

**Authors:** Changfeng Xiao, Xin Li, Zhizhao Ding, Hongcai Zhang, Wenwei Lv, Changsuo Yang, Daqian He, Lihui Zhu

**Affiliations:** 1 Institute of Animal Husbandry and Veterinary Science, Shanghai Academy of Agricultural Sciences, Shanghai 201106, China; xcf1234567@163.com (C.X.); lixinxinli_sadu@outlook.com (X.L.); dzz80557472@163.com (Z.D.); 13818518467@163.com (W.L.); yangchangsuo@189.cn (C.Y.); daqianhe@aliyun.com (D.H.); 2 School of Agriculture and Biology, Shanghai Jiao Tong University, Shanghai 200240, China; hczhang@sjtu.edu.cn

**Keywords:** fermented feed, microbiome, metabolome, squabs, gut

## Abstract

**Simple Summary:**

Rapeseed meal, soybean meal, and corn bran are significant sources of protein that could serve as alternatives to conventional protein sources in poultry production. Nonetheless, their utilization in pigeon production is limited by the presence of anti-nutritional factors in these plant materials, as well as the feeding preferences of pigeons, which tend to favor raw grains of corn, soybean, and pea. The microbial fermentation of feed breaks down anti-nutritional substances, and has attracted the attention of poultry producers due to its beneficial effects on growth performance and gut function. In this study, we treated pigeons with fermented mixed feed (soybean meal–rapeseed meal–corn bran (6:3:1, *m*/*m*/*m*)) to improve the growth performance and gut health of squabs. Our results found that the fermented mixed feed promoted the growth performance of squabs, enhanced their intestinal morphology, increased the relative abundance of beneficial bacteria, and decreased the relative abundance of harmful bacteria. Furthermore, the squabs’ metabolites were also influenced by the addition of the fermented feed. Overall, this study provides valuable insights into the potential benefits of using fermented feed in pigeon farming.

**Abstract:**

The purpose of this study was to evaluate the effect of fermented mixed feed (FMF) (soybean meal–rapeseed meal–corn bran (6:3:1, *m*/*m*/*m*)) on the growth performance, intestinal microbial communities, and metabolomes of squabs. One hundred and eighty 1-day-old squabs were randomly allocated to two groups, each containing six replicates of fifteen squabs cared for by 60 pairs of breeding pigeons secreting crop milk. Each pair of breeding pigeons cared for three squabs. The control group was fed a basal diet, while the experimental group was fed the basal diet containing 5% FMF. The results showed that daily weight gain, carcass weight, villus height, and the mRNA level of *ZO-1* in the ileum were increased in the birds fed FMF compared to the control squabs (*p* < 0.05). Greater abundances of beneficial bacteria such as *Lactobacillus*, *Bifidobacteria*, and *Bacillus* as well as fewer harmful bacteria (i.e., *Enterococcus*, *Veillonella*, and *Corynebacterium*) in the ilea of squabs fed FMF. Six differential metabolites were identified in the FMF-treated squabs; one metabolite was increased (ω-salicoyisalicin) and five were decreased (3-benzoyloxy-6-oxo-12-ursen-28-oic acid, estradiol-17-phenylpropionate, aminotriazole, phosphatidyl ethanolamine (22:6/0:0), and 1-arachidonoylglycerophosphoinositol). Positive correlations were observed between the abundance of *Lactobacillus* and villus height. Overall, FMF treatment improved both growth and intestinal health in pigeons, suggesting potential benefits for pigeon production.

## 1. Introduction

Compared to other poultry, pigeon (*Columba livia*) meat is valuable for humans due to its high nutritional value, low cholesterol content, and high protein content [1,2]. In China, pigeon farming represents the fourth largest fraction of the poultry industry after chicken, duck, and goose farming. Pigeon squabs are market-ready when they reach about 500 g (at about 25 days old) [3]. The health and growth performance of pigeons are closely related to the economic benefits of agricultural enterprises. Pigeons are late-maturing birds and rely on their parents for food as they gradually adapt to feeding independently. Parent pigeons secrete milk from crop epithelial cells to feed squabs [4]. This milk is composed of protein and fat; it is important for squabs that have just emerged from the shell as it is their only food source [5,6]. Therefore, the growth performance of squabs can be improved by adjusting the parental diet.

Corn, soybean, and pea are expensive plant-based protein materials widely used in pigeon feed due to pigeons’ preference for raw grains. The scarcity of these materials and their high cost are important factors restricting the economic benefits of poultry husbandry. The reasonable utilization of low-cost alternative feed is a potential solution to this problem [7]. Rapeseed meal and soybean meal are important sources of protein and are widely used in animal husbandry [8]. Similarly, corn bran is a byproduct of corn milling, which is rich in protein and hemicellulose, making it a potential protein alternative for use in poultry production [9]. However, the application of these alternative protein sources in poultry production is hindered by the many anti-nutritional factors they contain, such as glucosinolates, tannins, and phytic acid. Moreover, there is evidence that a diet high in rapeseed meal impairs intestinal function [10]. Since increased poultry performance is strongly associated with changes in gut function and the microbiome [11], maintaining intestinal health and alleviating intestinal inflammation are important issues for poultry production.

Microbial fermented feed contains beneficial microorganisms that break down anti-nutritional substances and induce more active probiotics, beneficial metabolites, soluble peptides, and other small molecules. This improves digestion, immunity, intestinal morphology, and animal gut microbiota, leading to increased production in poultry farming [12,13,14]. However, information on the effects of microbial fermented feeds on growth and gut health in squabs is still limited. 

This study aims to assess the impact of fermented mixed feed (FMF) on the growth performance, gut morphology, microbial abundance, and metabolites of squabs. This research seeks to validate the hypothesis that utilizing FMF consisting of rapeseed meal, soybean meal, and corn bran can enhance growth performance and promote intestinal morphology in squabs through changes in the gut microbiota and metabolism. Our study may provide a basis for the application of FMF in pigeons.

## 2. Materials and Methods

### 2.1. Preparation of FMF 

In this study, *Lactobacillus plantarum* and *Bacillus subtilis* were obtained from Beijing Xiecheng Biotechnology Co., Ltd. (Beijing, China) and used to ferment feed materials (corn bran, soybean meal, and rapeseed meal). To ferment the feed, a base matrix consisting of 60% soybean meal, 30% rapeseed meal, and 10% corn bran was used. A mixture of 1 kg of *Lactobacillus plantarum* powder (1.0 × 10^10^ CFU/g) and 1 kg of *Bacillus subtilis* powder (2 × 10^11^ CFU/g) was inoculated into 1000 kg of wet mixed medium. Sterile water was added with continuous mixing to achieve a system humidity of 40%. Subsequently, the moist substrate was placed in a plastic bag with a one-way valve and incubated for 14 days at 30 °C. Post fermentation, the pH of the substrate dropped below 6.0, and the lactic acid bacteria count exceeded 2 × 10^9^ CFU/kg of feed. The glucosinolates in the FMF were analyzed via chromatography, using a protocol indicated by Boege et al. [15]. The contents of phytic acid and lactic acid were measured using kits from Shanghai Macklin Biochemical Technology Co., Ltd. (Shanghai, China), following the manufacturer’s instructions. The nutritional value and chemical composition of the FMF are shown in Table S1.

### 2.2. Experimental Design

Sixty pairs of Carnean pigeons from Shanghai Pangji Pigeon Industry Co., Ltd. (Shanghai, China) demonstrating similar egg-laying performance were selected and allocated to two treatments: a control group (basal diet) and an FMF-treated group (basal diet + 5% FMF). There were six replicates and each replicate contained five pairs of nesting pigeons. Each pair of parent pigeons raised three 1-day-old squabs (90 squabs per group) in an artificial aviary equipped with three-layer cages that included perches and nests. Replicate samples were evenly distributed on the top, middle, and bottom floors of the cage to minimize the impact of cage position. The pigeons were fed the experimental diets for 43 days, including 18 days for breeding squabs and 25 days for nesting. The squabs were fed milk secreted by the pigeons in a beak-to-beak manner. The pigeons in the control group were fed the basic diet, which consisted of 66% raw grain and 34% pelleted feed. The pigeons in the FMF-treated group were fed a diet supplemented with 5% FMF to replace soybean in the raw grain in equal proportion. The composition and nutritional value of the basal diet are shown in Table S2. The pelleted feed used in this experiment measured 4 mm in diameter and 3–8 mm in length. As pigeons are known for their selective eating habits and tendency to play with food, the birds were fed artificially twice per day at 05:00 and 13:00 to minimize feed wastage. Three squabs from each replicate (fifteen squabs per treatment) were randomly selected and euthanized using pentobarbital sodium at 25 days of age. The anterior position of the ileum was sampled, and its contents were collected. The squabs were weighed on an empty stomach at 1 and 25 days of age, respectively. The animal study protocol was approved by the Ethics and Animal Welfare Committee of the Shanghai Academy of Agricultural Sciences (No. SAASPZ0521018).

### 2.3. Real-Time Quantitative PCR (RT-qPCR)

Ileal samples (*n* = 12/group) weighing approximately 100 mg were collected and quickly frozen in liquid nitrogen for an RT-qPCR analysis. Total RNA was extracted from ileal samples using TRIzol reagent (Life Technologies, Carlsbad, CA, USA) and reverse-transcribed using a PrimeScript RT kit (DRR047A, Takara, Japan). The RT-qPCR was performed on an ABI Quant Studio™ 5 (Applied Biosystems, Foster City, CA, USA) with a total reaction mix volume of 20 μL, using Hieff^®^ qPCR SYBR Green Master Mix (Thermo Fisher Scientific, Waltham, MA, USA) according to the manufacturer’s instructions. The primers used in this study are shown in Table S3. β-actin was used as an endogenous reference. The 2-ΔCt value was used to represent the expression of each gene [16]. 

### 2.4. Histopathological Analysis 

Ileal samples (*n* = 12/group) measuring approximately 2 cm × 2 cm were cut and immersed in 4% formaldehyde for histological analysis. Subsequently, they were stained with hematoxylin and eosin (H&E) and examined via Olympus light microscopy (Tokyo, Japan). We calculated the villus height, villus width, crypt depth, and the ratio of villus height to crypt depth (VCR). The samples were also examined using transmission electron microscopy ((TEM) Hitachi H-7600, Tokyo, Japan), as described previously [17]. 

### 2.5. Gut Microbiome and Metabolomic Analyses 

16S rRNA sequencing and a metabolomic analysis were performed by Shanghai Meiji Biotechnology Co., Ltd. (Shanghai, China). Microbiome and metabolome analyses were performed on the Majorbio I-Sanger Cloud Platform (https://cloud.majorbio.com/; accessed on 18 August 2023). Briefly, for 16S rRNA sequencing, microbial genomic DNA was extracted from the ileal contents. The V3–V4 variable region of the 16S rRNA was amplified using its universal primer sequence 338F, 5′-ACTCCTACGGGAGGCAGCAG-3′, and 806R, 5′-GGACTACHVGGGTWTCTAAT-3′. The PCR products from the control (*n* = 12) and fermented (*n* = 12) groups were sequenced using an Illumina MiSeq PE300 platform (San Diego, CA, USA). Quality control and filtering of the raw FASTQ sequences were performed using DADA2 (https://github.com/benjjneb/dada2 accessed on 18 August 2023) software to obtain representative sequences of the amplicon sequence variant representative sequences. Raw FASTQ sequences were filtered first for reads with adapter contamination at the end of the read, reads < 50 bp, and reads with a quality score < 20 were removed using the Trimmomatic program. Taxonomic assignment, alpha diversity, beta diversity, and species difference analyses were investigated using QIIME. Four individuals were excluded from our analysis based on the results of a principal component analysis (PCA), non-metric multidimensional scaling (NMDS), and a principal coordination analysis (PCoA); therefore, nine individuals from the control group and 11 individuals from the FMF-treated group were selected for further analysis. 

For the metabolomic analysis, metabolites were extracted from the ileal contents using 400 μL of a methanol–water (1:1, *v*/*v*) solution and ultra-high-performance liquid chromatography–mass spectrometry (UHPLC-MS). UHPLC-MS was conducted on a UHPLC-Q Exactive system (Thermo Fisher Scientific), using an HSS T3 column (100 mm × 2.1 mm, 1.8 μm; Waters, Milford, MA, USA) coupled with a Thermo UHPLC-Q Exactive Mass Spectrometer equipped with an electrospray ionization source operating in positive or negative ion mode. The metabolites were identified by searching the Human Metabolome Database (http://www.hmdb.ca/ accessed on 18 August 2023) and the Metlin (https://metlin.scripps.edu/ accessed on 18 August 2023) database. Metabolic features detected in at least 80% of any set of samples were retained. A PCA and a partial least squares discriminant (PLS-DA) analysis were performed using the R package “ropls” (Version 1.6.2) to evaluate the stability of the model. Different metabolites with a variable importance in projection (VIP) score > 1, *p* < 0.05, were determined using the PLS-DA model and the *p*-value generated via Student’s *t*-test. Differential metabolites were mapped onto their biochemical pathways through metabolic enrichment and pathway analyses based on the KEGG database (http://www.genome.jp/kegg/ accessed on 18 August 2023). The Python package “scipy.stats” (https://docs.scipy.org/doc/scipy/ accessed on 18 August 2023) was used to perform the enrichment analysis to obtain the most relevant biological pathways for experimental treatments.

### 2.6. Statistical Analyses

All data were expressed as mean ± SD values and analyzed using GraphPad Prism 8.0 software (San Diego, CA, USA). Significant differences in growth, gut morphology parameters, and metabolites between groups were determined using Student’s *t* test. The Wilcoxon rank-sum test was used to test for between-group differences in diversity indices and to screen for differential microbiota; after correcting for the false discovery rate, *p* < 0.05 was considered to indicate a significant difference. A Spearman correlation analysis was conducted to analyze the relationships among gut morphology parameters, microbiota, and metabolites; *p* < 0.05 was deemed statistically significant.

## 3. Results

### 3.1. Growth and Carcass Performance of Squabs

As shown in Table 1, the FMF significantly improved the carcass performance of the squabs, including their carcass weight (*p* < 0.01) and chest muscle weight (*p* < 0.05). In addition, the body weight and average daily gain (ADG) of the squabs were higher in the FMF-treated group than in the control group (*p* < 0.01).

### 3.2. H&E Staining, qRT-PCR, and TEM

**Figure 1:**
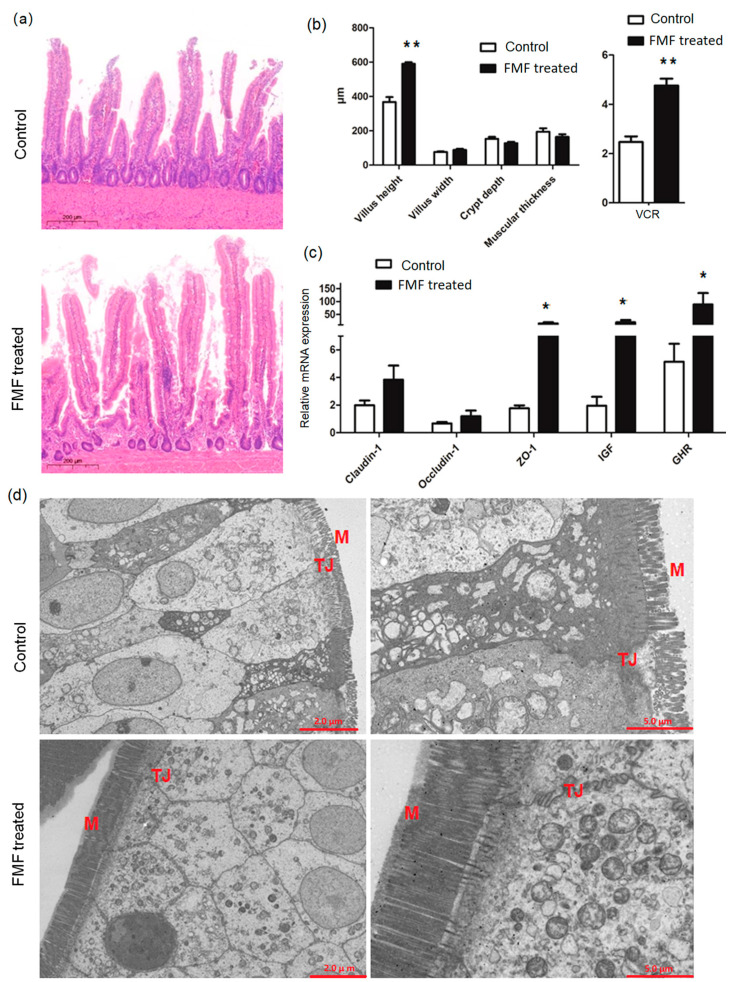
Dietary supplementation with fermented feed improved the intestinal function of squabs. (**a**) A histopathological section of the ileum stained using hematoxylin and eosin and visualized at 200 um. Tissue samples were collected at 25 days of age and were fixed in formalin for routine H&E staining. The section is representative of 12 samples from each group. (**b**) Morphological measurements of the ileum. Data are presented as the mean ± SD values of each group (*n* = 12). (**c**) The effects of dietary supplementation with fermented feed on the mRNA expression of tight junction proteins in the ilea of squabs. Data are presented as the mean ± SD values of each group (*n* = 12). (**d**) Transmission electron microscopy analysis of the ileum. The section is representative of 6 samples from each group. Data were analyzed using Student’s *t*-test, and *p*-values were considered statistically significant at * *p* < 0.05 and ** *p* < 0.01. VCR, the ratio of villus height to crypt depth; M, microvilli; TJ, tight junction; IGF-1, insulin-like growth factor-1; GHR, growth hormone receptor; FMF, fermented mixed feed; H&E, hematoxylin and eosin.

The diet containing FMF increased the VCR of the squabs (*p* < 0.01) and decreased crypt depth (*p* = 0.03) compared to the controls, but there was no obvious difference in villus height and width, muscle layer thickness, or villi between the two groups (*p* > 0.05) (Figure 1a,b). The expression levels of insulin-like growth factor-1 (*IGF-1*), growth hormone receptor (*GHR*), and *ZO-1* in the intestines of FMF-fed squabs were increased (*p* < 0.01) compared with the control squabs, but the expression levels of *Occludin-1* and *Claudin-*1 were unchanged (*p* > 0.05) (Figure 1c). TEM showed that the ileal microvilli in the control group were thick, short, and disordered, showing breakage, and the tight junctions of epithelial cells were disconnected (Figure 1d). However, the intestinal microvilli of the FMF-fed squabs were long and fine, the tight junctions between epithelial cells were intact, and locking proteins were visible in the ileum. The gaps between cells were small, and the number of mitochondria was large (Figure 1d).

### 3.3. Taxonomic Analysis

**Figure 2:**
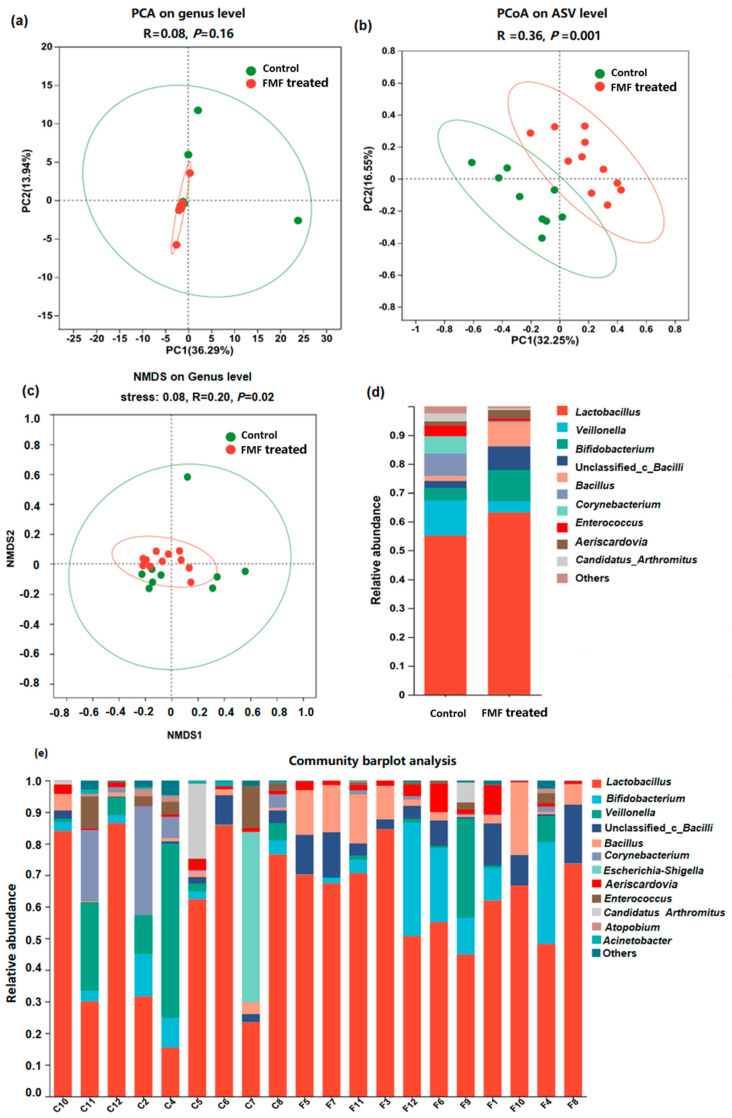
Bacterial composition and diversity were analyzed between the control and FMF−treated groups. (**a**) Principal component analysis (PCA). (**b**) Principal coordinate (PCoA) analysis. (**c**) Non-metric multidimensional scaling (NMDS) analysis. (**d**) Distribution of taxa between control and FMF-treated squabs at 25 days of age (each color represents one bacterial genus). Mean phylum-level relative abundances as detected via 16S rRNA sequencing. (**e**) Microbial relative abundance at the genus level for the control and FMF-treated groups. The contents of the squabs’ ilea were collected at 25 days of age. Nine samples from the control group and eleven samples from the FMF-treated group were used for bacterial composition and diversity analyses. C: control; F: FMF-treated group. Data were analyzed using the Wilcoxon rank-sum test, and *p*-values were considered statistically significant at *p* < 0.05 and *p* < 0.01.

**Figure 3:**
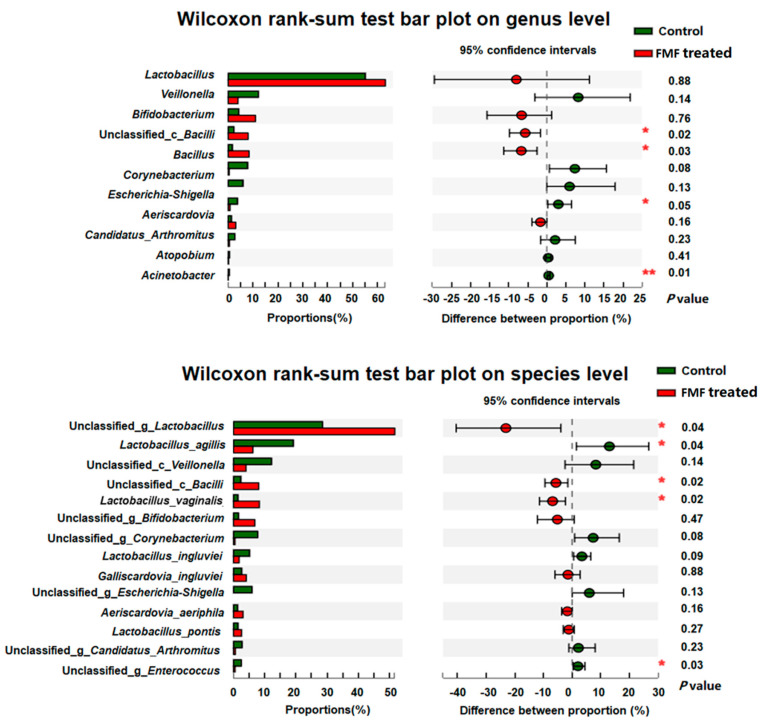
Bacterial genera and species differed between the control and FMF-treated groups. The contents of the squabs’ ilea were collected at 25 days of age. Nine samples from the control group and eleven samples from the FMF-treated group were used for genus and species difference analyses. Data were analyzed using the Wilcoxon rank-sum test, and *p*-values were considered statistically significant at * *p* < 0.05 and ** *p* < 0.01.

In both groups of squabs, the coverage index of the depth of the 16S rDNA sequencing of intestinal microorganisms was >95% (Table S4). The Sobs and Chao indexes were lower in the FMF-treated squabs than in the control squabs (*p* = 0.04), whereas differences in the Shannon and Simpson indexes were not significant (*p* > 0.05). PCoA can reflect the degree of difference in a sample’s community composition through the distance between points, the closer the distance between the points of the sample, the smaller the difference. NMDS and PCoA analyses indicated a clear separation between the FMF and control groups (Figure 2a–c) (*p* < 0.01). At the genus level (Figure 2d,e and Figure S1), the abundances of *Lactobacillus*, *Bifidobacterium*, *Bacillus*, and unclassified_c_*Bacilli* in the ilea of the FMF-fed squabs increased from 55.12% to 63.10%, from 4.32% to 10.93%, from 2.38% to 8.05%, and from 1.74% to 8.43%, respectively, compared to the control birds. The proportions of *Enterococcus*, *Veillonella*, *Corynebacterium*, and *Candidatus Arthromitus* decreased from 3.77% to 0.73%, from 12.23% to 3.98%, from 7.85% to 0.48%, and from 2.75% to 0.57%, respectively. At the genus level, *Bifidobacterium* and *Bacillus* showed higher abundances (*p* < 0.05) and *Enterococcus* and *Acinetobacter* showed lower abundances (*p* < 0.05) in the FMF-treated squabs compared with the control squabs (Figure 3a). At the species level, the FMF significantly increased the abundance of unclassified_g_*Lactobacillus*, unclassified_c_*Bacilli*, and *Lactobacillus_vaginalis* but decreased the abundance of *Lactobacillus agilis* and unclassified_g_*Enterococcus* in the ilea of the squabs (*p* < 0.05) (Figure 3b).

### 3.4. Metabolite Analysis

**Figure 4:**
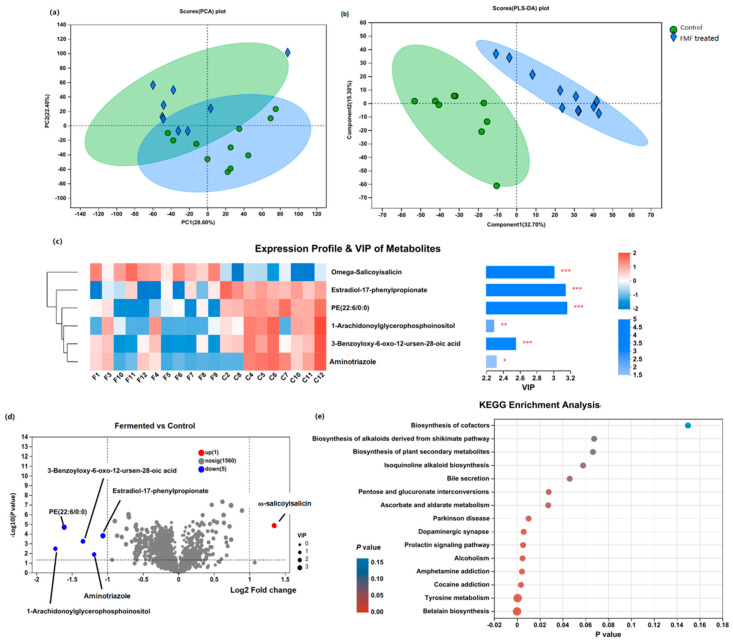
Metabolomic data profiles and pathway enrichment analysis. (**a**) Principal component analysis (PCA). (**b**) Partial least squares discriminant analysis (PLS−DA). (**c**) Z-score heatmap of 6 significantly altered metabolites determined using VIP scores from pairwise PLS-DA analysis with VIP > 1, *p* < 0.05, and fold change > 2 as cut-off values for significance. (**d**) Volcano plot of altered metabolites. (**e**) Pathway enrichment analysis of 34 significantly altered metabolites with VIP > 1, *p* < 0.05, and fold change > 1.5. Contents of squabs’ ilea were collected at 25 days of age. Nine samples from control group and eleven samples from FMF group were used for metabolomics analysis. Data were analyzed using Student’s *t* test, and *p*-values were considered statistically significant at * *p* < 0.05, ** *p* < 0.01, and *** *p* < 0.001. PE (22:6/0:0), phosphatidyl ethanolamine (22:6/0:0). C: control; F: FMF-treated group.

Overall, 833 annotated metabolites were identified in the ilea of the two groups, including 429 (40.62%) lipids and lipid-like molecules, 269 (25.47%) organic acids and derivatives, 114 (10.80%) organic oxygen compounds, and 103 (9.75%) organoheterocyclic compounds (Figure S2). A score plot of the PCA results showed that PC1 and PC2 explained 27.50% and 21.60% of the total variance, respectively (Figure 4a). The PLS-DA revealed that samples from the FMF group were distinguishable from those from the control group (Figure 4b). Six differential metabolites were screened via the PLS-DA (VIP > 1; *p* < 0.05; fold change > 2), including one upregulated metabolite (ω-salicoyisalicin) and four decreased metabolites (estradiol-17-phenylpropionate, aminotriazole, 3-benzoyloxy-6-oxo-12-ursen-28-oic acid, phosphatidyl ethanolamine (PE, 22:6/0:0), and 1-arachidonoylglycerophosphoinositol) in the FMF group (Figure 4c,d and Table S5). A KEGG analysis of the 34 differential metabolites (Table S5) screened via the PLS-DA (fold change > 1.5) showed that these altered metabolites were enriched in the betalain biosynthesis, tyrosine metabolism, cocaine addiction, and ascorbate and aldarate metabolism pathways (Figure 4e).

### 3.5. Analysis of Correlations between Metabolites and Microorganisms

**Figure 5:**
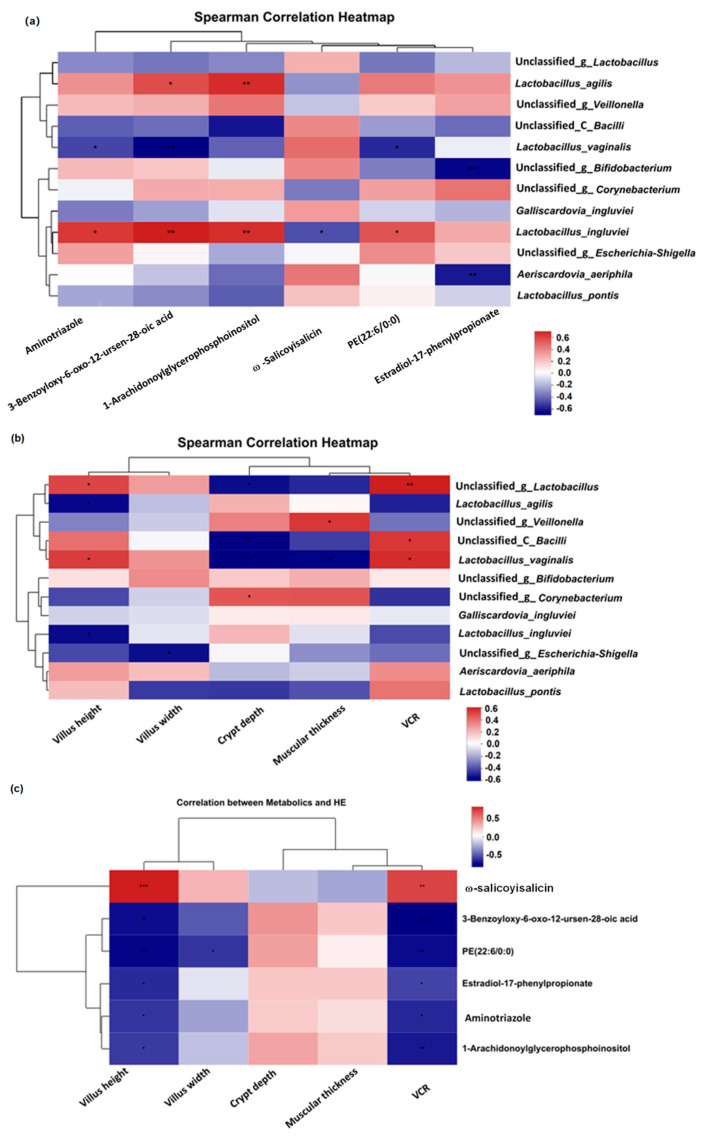
Enhanced intestinal function was associated with metabolites and the microbiome. The heatmap depicts the associations between (**a**) taxa and metabolites, (**b**) taxa and intestinal morphology, and (**c**) metabolites and intestinal morphology that differed in the FMF-treated group. Correlations of two variables with *p* < 0.05 are shown. The contents of the squabs’ ilea were collected at 25 days of age. Nine samples from the control group and eleven samples from the FMF-treated group were used for the correlation analysis. Data were analyzed using a Spearman correlation analysis, and *p*-values were considered statistically significant at * *p* < 0.05, ** *p* < 0.01, and *** *p* < 0.001. Red represents positive correlations, and blue represents negative correlations. VCR, the ratio of villus height to crypt depth; PE (22:6/0:0), phosphatidyl ethanolamine (22:6/0:0).

As shown in Figure 5a, the relative abundance of *Lactobacillus_vaginalis*_g_*Bacillus* demonstrated negative correlations with the contents of aminotriazole, PE (22:6/0:0) and 3-benzoyloxy-6-oxo-12-ursen-28-oic acid, while the abundance of *Lactobacillus ingluviei* was positively correlated. In addition, unclassified_g_*Bifidobacterium* and *Aeriscardovia aeriphila* were negatively correlated with estradiol-17-phenylpropionate, ω-Salicoyisalicin, and *L. ingluviei*. Furthermore, positive correlations were found between the abundance of *Lactobacillus_vaginalis* and villus height and VCR; positive correlations were also found between the abundances of unclassified_g_*Lactobacillus* and unclassified_c_*Bacilli* and VCR (Figure 5b). The contents of aminotriazole, PE (22:6/0:0), estradiol-17-phenylpropionate, and 1-arachidonoylglycerophosphoinositol were negatively associated with villus height and VCR, while ω-salicoyisalicin was positively correlated with villus height and VCR (Figure 5c). A negative correlation was also found between villus width and PE (22:6/0:0).

## 4. Discussion

Corn, soybean, and pea are the main sources of protein for pigeons due to pigeons’ habits. With increases in the cost of these materials, there is an urgent need to find suitable protein alternatives for pigeon production. Rapeseed meal, soybean meal, and corn bran are byproducts of the food industry, which are rich in protein and demonstrate well-balanced amino acid compositions; their use in poultry production has been considered. However, their application in pigeon production is limited in China because they contain anti-nutritional factors which may negatively affect the poultry industry [10]. Microbial fermentation is a low-cost method developed in recent years to enhance the nutritional quality of livestock and poultry feed to promote gut health and productivity [12,14,18]. In previous research, fermented rapeseed meal and soybean meal were included in the diets of broiler chickens and laying hens [19,20,21], improving production performance and keeping the birds in good health. In the present study, we found that the inclusion of FMF in pigeon feed increased squabs’ growth performance and enhanced gut function. Changes identified in the microbiome were strongly correlated with the metabolome and morphological characteristics, suggesting the feasibility of partially replacing soybean with fermented feed in pigeon production. 

The quality of intestinal function is closely related to the production performance of poultry. Intestinal villi are the main actors in the small intestine; they are circular folds formed by the inward protrusion of the intestinal wall. It is generally believed that a lower crypt depth, greater villus height, and increased VCR reflect better intestinal structure and function, which can promote the digestion and absorption of nutrients and improve disease resistance and growth performance [22]. Maintaining normal gut function is critical to animal health and productivity. However, as squabs mature, their intestines face many stresses, including those due to their environment, nutrition, antibiotics, and diseases that affect intestinal function, digestion, and absorption [23,24,25]. Occludin, claudin-1, and ZO-1 maintain intestinal integrity by forming boundary junctions between adjacent cells. Intestinal permeability is primarily regulated by tight junctions. Here, we observed decreases in crypt depth but increases in the VCR and mRNA levels of *IGF-1*, *GHR*, and *ZO-1* in the ileal tissues of squabs whose parents were fed FMF for 43 days. This demonstrates that FMF can improve the gut morphology of squabs and help promote gut functions such as digestion and the absorption of nutrients. These results were consistent with those of studies conducted on broiler chickens and laying hens, which demonstrated that fermented feed improved gut villi morphology and immune responses [12,19,26].

Gut bacteria play important roles in animal health, including food digestion, energy production, and immune regulation and resistance [27]. Dietary composition could affect the gut epithelial barrier and microbial community [28]. In our study, the addition of FMF to a base diet influenced the alpha diversity and beta diversity of the squabs’ intestinal microbiota, indicating that FMF may influence species taxa and abundance. *Lactobacillus*, *Bifidobacterium*, and *Bacillus* are all genera of bacteria that play important roles in human and animal health, especially in relation to improving intestinal barrier function. The abundances of these probiotic genera were increased in the ilea of the FMF-treated squabs, while the potentially pathogenic bacteria *Enterococcus*, *Veillonella*, *Corynebacterium*, and *Candidatus Arthromitus* were less abundant. Several strains of *Lactobacillus* and *Bifidobacterium* have been shown to help eliminate pathogenic bacteria [29] and improve intestinal health [30], immune regulation [31,32], and metabolic regulation [33]. *B. subtilis* was also shown to benefit gut function by modulating gut microbiota in laying hens [34]. An increased abundance of *Candidatus Arthromitus* was associated with depression [35]. *Corynebacterium* spp. are pathogens associated with orthopedic infections and contact with infected cattle [36,37]. In this study, the abundances of the genera *Lactobacillus* and *Bifidobacterium* were increased, while the abundance of the genus *Corynebacterium* was decreased in the FMF-treated squabs; these results are consistent with the results found in broilers fed fermented feed [14]. Additionally, changes in gut morphology observed in the context of diet correlate well with concurrent observations of changes in gut microbiota [38]. Here, we observed that the enrichment of *Lactobacillus* and *Bacillus* species was positively associated with villus height and VCR, suggesting that these bacteria have a positive effect on squab gut health and further supporting the morphological changes found in the FMF-treated group. 

There is a close relationship between gut function, fatty acids, and gut microbes [39,40]. To better understand the beneficial effects of FMF on gut health, we analyzed the production of metabolites in the ileal contents of squabs. Our metabolic profiling revealed that 34 metabolites changed in the FMF-treated group, which was screened via PLS-DA (fold change > 1.5). Arginine, which was enriched in the fermented-feed-treated squabs, has been reported to be an important amino acid for poultry, especially those under stress [41]. Arginine participates in the restoration of intestinal epithelial cells [42], and L-arginine can reduce intestinal mucosal injury induced by *Clostridium perfringens* colonization in broiler chickens [43]. Additionally, the contents of 1-arachidonoylglycerophosphoinositol, estradiol-17-phenylpropionate, aminotriazole, 3-benzoyloxy-6-oxo-12-ursen-28-oic acid, and PE (22:6/0:0) were decreased in the FMF group. However, the functions of these metabolites are still unclear and require further consideration. We also found that the enrichment of *Lactobacillus_vaginalis* was negatively correlated with low contents of aminotriazole, PE (22:6/0:0), and 3-benzoyloxy-6-oxo-12-ursen-28-oic acid. The abundances of unclassified_g_*Bifidobacterium* and *A. aeriphila* were negatively correlated with estradiol-17-phenylpropionate. This suggests that interactions between these altered metabolites and microbes play a potential role in regulating intestinal functional in squabs fed a diet supplemented with FMF.

A pathway enrichment analysis showed that betalain biosynthesis, tyrosine metabolism, the prolactin signaling pathway, dopaminergic synapses, and ascorbate and aldarate metabolism were altered in the FMF-treated squabs compared with the controls. The prolactin signaling pathway needs prolactin, a hormone that is essential for normal reproduction and sexual behavior [44,45]. Ascorbate and aldarate metabolism were also reported to be positively associated with the increased abundance of *Prevotellaceae* UCG-004 in Hu sheep fed silage mixed with Chinese cabbage [46]. *Prevotellaceae* produce butyrate, which plays an important role in gut barrier integrity and homeostasis [47]. Gut microbes play active roles in the metabolism of serum inositol, tyrosine, and glycine [48]. A high serum level of tyrosine might be a useful indicator of severely compromised intestinal villi [48]. The alterations in tyrosine metabolism, the prolactin signaling pathway, and ascorbate and aldarate metabolism observed in this study may be due to alterations in the colonization of the gastrointestinal microbiota.

Additionally, complex interactions and interdependencies between gut morphology, gut microbiota, and metabolites collectively impact host health and disease development. Evidence has indicated that the composition of gut microbiota and metabolic activity are influenced by gut morphology, while metabolites can act as signaling molecules for host physiological responses, influencing gut morphology and function [49,50]. The length and surface area of intestinal villi provide various attachment sites and affect nutrient absorption efficiency for microorganisms. On the other hand, microorganisms regulate morphology through their metabolic activity, with beneficial microorganisms helping to maintain intestinal barrier integrity and promote epithelial cell proliferation and differentiation [49]. Changes in microbial communities can alter metabolite production, subsequently influencing intestinal morphology and overall health. Metabolites produced by gut microbiota, short-chain fatty acids in particular, have been shown to potentially enhance intestinal morphology [50]. Here, villus height and VCR were positively correlated with the genera *Lactobacillus* but negatively associated with the contents of aminotriazole, PE (22:6/0:0), estradiol-17-phenylpropionate, and 1-arachidonoylglycerophosphoinositol, indicating that the function of FMF in promoting the development of intestinal villi may be related to changes in these probiotics and metabolites. 

There were several limitations in our study. First, the study mainly focused on fermented diet-associated microbiome and metabolome changes, and the functions of the metabolites changed must be investigated further. Second, pigeons are considered altricial birds. Squabs rely on their parents for food since they are unable to feed themselves. Methods of effectively measuring roast squabs’ feed intake and feed conversion efficiency are controversial. Therefore, this study only focused on measuring the growth and carcass performance of squabs while ignoring the consumption of feed by breeding pigeons to maintain their own metabolism. Finally, since FMF is fed to squabs by their parents, the maintenance of intestinal health through the use of fermented feed may provide the same benefits in breeding pigeons, a hypothesis which requires further investigation.

## 5. Conclusions

Our results showed that FMF promoted growth performance, enhanced intestinal health, and regulated the microbiota of squabs by reducing the abundance of pathogenic microorganisms, including *Enterococcus*, *Veillonella*, *Corynebacterium*, and *Candidatus Arthromitus,* and improving the abundance of beneficial microorganisms like *Lactobacillus*, *Bifidobacterium*, and *Bacillus*. We also found that the ileal microbiome of the FMF-treated squabs correlated with their metabolites and intestinal morphology, emphasizing a plausible mechanism which requires further investigation. Further research is also required on the optimization of the fermentation process and the appropriate application of FMF in pigeon nutrition to make the final product economically feasible.

## Figures and Tables

**Table 1:** Growth and carcass performance of squabs.

Item	Control	FMF
Initial body weight (g)	9.21 ± 0.46	9.55 ± 0.58
Final body weight (g)	478.27 ± 26.87 ^a^	562.33 ± 36.83 ^c^
ADG (g/d)	18.76 ± 1.07 ^a^	22.11 ± 1.46 ^b^
Final carcass weight (g)	404.13 ± 25.53 ^a^	495.53 ± 37.96 ^c^
Final chest muscle weight (g)	91.31 ± 13.35 ^a^	103.05 ± 9.61 ^b^

Different letters indicate significant differences compared to the control (Student’s *t* test; adjacent letters indicate *p* < 0.05 and spaced letters indicate *p* < 0.01). Results are shown as mean ± SD values. FMF, fermented mixed feed; ADG, average daily gain.

## Data Availability

Raw data of microbiome and metabolome are deposited at NCBI under the SRA database with accession No. PRJNA967978 and Metabo-Lights with accession No. MTBLS7803.

## Supplementary Materials

The following supporting information can be downloaded at: https://www.mdpi.com/article/10.3390/ani14101411/s1, Table S1: Chemical and anti-nutritional factor analysis of fermented mixed feed; Table S2: Composition and nutrition level of experimental diet (air dry basis); Table S3: Primers used for real-time quantitative PCR; Table S4: Comparison of microbial species abundance and diversity index of squabs; Table S5: Differentially expressed metabolites in squabs treated by fermented mixed feed; Figure S1: Microbial relative abundance at the genus level for control and FMF groups; Figure S2: Classification of metabolites.
