# Supplementary material for: Enhancing Growth and Gut Health in Squabs: The Impact of Fermented Mixed Feed
Source: Animals (Basel). 2024 May 8;14(10):1411. doi: 10.3390/ani14101411 (PMC11117316; doi:10.3390/ani14101411)
Supplement: Supplementary file 1 [file animals-14-01411-s001.zip › animals-2948688-supplementary.pdf]

Journal name: Animals

Enhancing Growth and Gut Health in Squabs: The Impact of Fermented Mixed Feed

Changfeng Xiao [a†], Xin Li [a†], Zhizhao Ding [a], Wenwei Lv [a], Changsuo Yang [a], Daqian He [a], Lihui Zhu [a*]

[a] *Institute of Animal Husbandry and Veterinary Science, Shanghai Academy of Agricultural Sciences, Shanghai 201106*

[†]These authors contributed equally to this work.

[*]Corresponding author: Lihui Zhu. Email: zhulihui@saas.sh.cn. Address: Shanghai Academy of Agricultural Sciences; 2901 Beidi Road, Shanghai, China.

Table S1 Chemical and anti-nutritional factor analysis of fermented mixed feed

Item	Un-fermented	Fermented
Crude protein (%)	38.13	41.8
Crude fat (%)	3.71	5.00
Crude fiber (%)	7.24	6.95
Phytic acid (mg/g)	7.99	7.07
Lactic acid, (mg/g)	6.18	23.46
Glucosinolates (μmol/g)	10.72	7.37

Table S2 Composition and nutrition level of experimental diet (air dry basis)

Ingredients	Content (%)		Nutrient level[1)]	Content (%)	
	Basal diet	Basal diet + 5% FMF		Content (%)	Basal diet + 5% FMF
**Raw Grain**			ME(MJ/kg)	12.08	12.07
Corn	26.4	26.4	Crude protein	15.41	15.42
Soybean	19.8	14.8	Calcium	2.42	2.43
Wheat	16.5	16.5	Lysine	0.73	0.72
Sorghum	3.3	3.3	Methionine	0.41	0.42
**Pelleted feed**					
Corn	10.88	10.88			
Soybean	4.08	4.08			
Wheat	1.02	1.02			
Soybean meal	11.22	11.22			
Wheat skin	0.51	0.51			
Wheat middlings	1.70	1.70			
Conch meal	0.34	0.34			
Limestone	0.34	0.34			
$CaHPO_4$	0.34	0.34			
Soya-bean oil	3.40	3.40			
Nacl	0.07	0.07			
Premix [2)]	0.10	0.10			
**FMF**	0.00	5			
**Total**	100	100			

[1)] The nutrient content was measured by the mixture of raw grain and pelleted feed except the ME. The experimental diets were formulated to contain similar levels of metabolic energy (ME) and crude protein (CP) to meet the nutritional needs of pigeons according to the NRC (Nutrient requirements of poultry. Washington, DC: The National Academies Press, 1994).

[2)] the premix provided the follows per kilogrm of diet VA 3,200 KIU, VD3 800 KIU, VE 15IU, VK 16mg， VB1 8mg, VB2 24.8 mg, Calcium pathtothenate 400 mg, niacin 32.5 mg, VB6 64mg, VB12 5mg, biotin 2mg, Fe 60 mg, Zn 66mg, Mn 65mg, Se 0.3 mg, I 1mg.

Table S3 Primers used for real-time quantitative PCR

Primers	F/R	5'→3'	No.of genes	Product （bp）
β-actin	F	CCCATCTACGAAGGCTACGC	XM_021291357.1	150
	R	CTTGATGTCACGCACAATTTC		
IGF-1	F	CCAATGGGCATTCTCCCAGT	XM_005500280.3	181
	R	TAGGCTTTTGAGGGGCACAG		
GHR	F	ACCCAGGCTCTCAACAATGG	NM_001282815.1	227
	R	CTGTTACGGCCAGACCACAT		
Occludin-1	F	GCTTCTACCTGGTGGTGATTG	XM_005509325.2	328
	R	TCCCAGTAGATGTTTTGTTTCC		
Claudin	F	AGCCTCATCTCCACCATCTAT	XM_021299640.1	325
	R	TGCCACTATTGTGATTTCCAG		
ZO-1	F	GAACCAAAGCCAGTGTATG	XM_021299314.1	247
	R	GGTCCCCTTCCTCTAATC		

Table S4 Comparison of microbial species abundance and diversity index of squabs

Items	Control	FMF	*P* value
Sobs index	22.33±14.76	12.09±6.04	0.04
Chao index	22.96±16.24	12.09±6.04	0.04
Shannon index	1.15±0.45	1.07±0.27	0.6
Simpson index	0.49±0.21	0.48±0.12	0.82
Coverage	＞0.95	＞0.95	—

Table S5 Differentially expressed metabolites in squabs treated by fermented mixed feed

Metabolite	Formula	Fold change (Fermented/Control)	*P*_value	FDR
Omega-Salicoyisalicin	C20H22O9	2.55	0.00	0.00
6-{3-[2,3-dioxo-3-(2,3,4,6-tetrahydroxyphenyl)propyl]-5-hydroxyphenoxy}-3,4,5-trihydroxyoxane-2-carboxylic acid	C21H20O14	1.86	0.00	0.00
Estrone-3,4-quinone	C18H20O3	1.67	0.00	0.01
Methionyl-Arginine	C11H23N5O3S	1.67	0.00	0.00
10-Acetoxyoleuropein	C27H34O15	1.65	0.00	0.00
(2xi,4xi)-4,4',5,7-Tetrahydroxyflavan	C15H14O5	1.62	0.00	0.01
L-Dopa	C9H11NO4	1.60	0.02	0.06
3-{[3,4-dihydroxy-5-(hydroxymethyl)oxolan-2-yl]oxy}-5,7-dihydroxy-2-(4-hydroxy-3-methoxyphenyl)-3,4-dihydro-2H-1-benzopyran-4-one	C21H22O11	1.60	0.00	0.01
Dopaquinone	C9H9NO4	1.59	0.00	0.00
7-CHLOROETHYLTHEOPHYLLINE	C9H11ClN4O2	1.56	0.00	0.00
Cymorcin diglucoside	C22H34O12	1.55	0.00	0.00
3,4,5-trihydroxy-6-[4-(5,7,8-trihydroxy-4-oxo-3,4-dihydro-2H-1-benzopyran-2-yl)phenoxy]oxane-2-carboxylic acid	C21H20O12	1.54	0.00	0.00
Gly Ile Gly Val	C15H28N4O5	0.67	0.00	0.02
17-hydroxyandrostane-3-glucuronide	C25H40O9	0.66	0.00	0.02
9'-Carboxy-gamma-chromanol	C23H36O4	0.66	0.00	0.00
Val-Ala-OH	C13H16N2O6	0.66	0.00	0.01
O-Arachidonoyl Glycidol	C23H36O3	0.66	0.00	0.00

2-(2,4-dihydroxy-5-methoxyphenyl)-3-(3,7-dimethylocta-2,6-dien-1-yl)-5,7-dihydroxy-6-(3-methylbut-2-en-1-yl)-4H-chromen-4-one	C31H36O7	0.66	0.04	0.11
(3'x,5'a,9'x,10'b)-O-(3-Hydroxy-6-oxo-7-drimen-11-yl)umbelliferone	C24H28O5	0.65	0.00	0.01
Glu Val Ile Glu	C21H36N4O9	0.65	0.01	0.04
2-benzyloctan-1-ol	C15H24O	0.65	0.01	0.04
2-(2,4-dihydroxyphenyl)-5,7-dihydroxy-3-(4-hydroxy-3,7-dimethylocta-2,6-dien-1-yl)-6-(3-methylbut-2-en-1-yl)-4H-chromen-4-one	C30H34O7	0.63	0.00	0.01
11'-Carboxy-gamma-tocotrienol	C25H36O4	0.62	0.00	0.00
5,8,12-Trihydroxy-9-octadecenoic acid	C18H34O5	0.61	0.00	0.00
PE(22:5/0:0)	C27H46NO7P	0.60	0.00	0.00
Val Leu His	C17H29N5O4	0.60	0.00	0.01
3,5-dihydroxy-2-[(2E)-3-(4-hydroxyphenyl)prop-2-enoyl]cyclohexa-2,5-diene-1,4-dione	C15H10O6	0.58	0.00	0.01
PE(16:1/0:0)	C21H42NO7P	0.55	0.00	0.00
LysoPE(22:6(4Z,7Z,10Z,13Z,16Z,19Z)/0:0)	C27H44NO7P	0.54	0.00	0.00
Estradiol-17-phenylpropionate	C27H32O3	0.48	0.00	0.00
Aminotriazole	C48H74O18	0.44	0.01	0.05
3-Benzoyloxy-6-oxo-12-ursen-28-oic acid	C37H50O5	0.39	0.00	0.01
PE(22:6/0:0)	C27H44NO7P	0.33	0.00	0.00
1-Arachidonoylglycerophosphoinositol	C29H49O12P	0.30	0.00	0.02

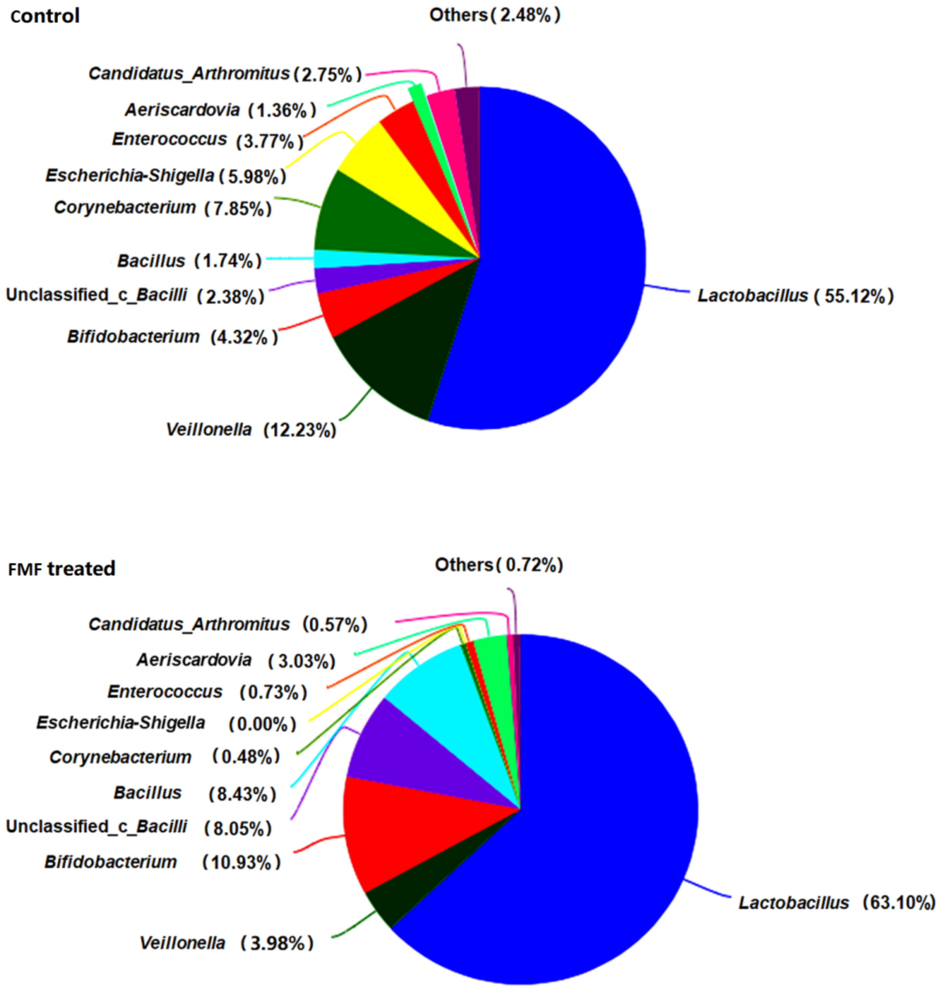

Figure S1 Microbial relative abundance at the genus level for control and FMF groups

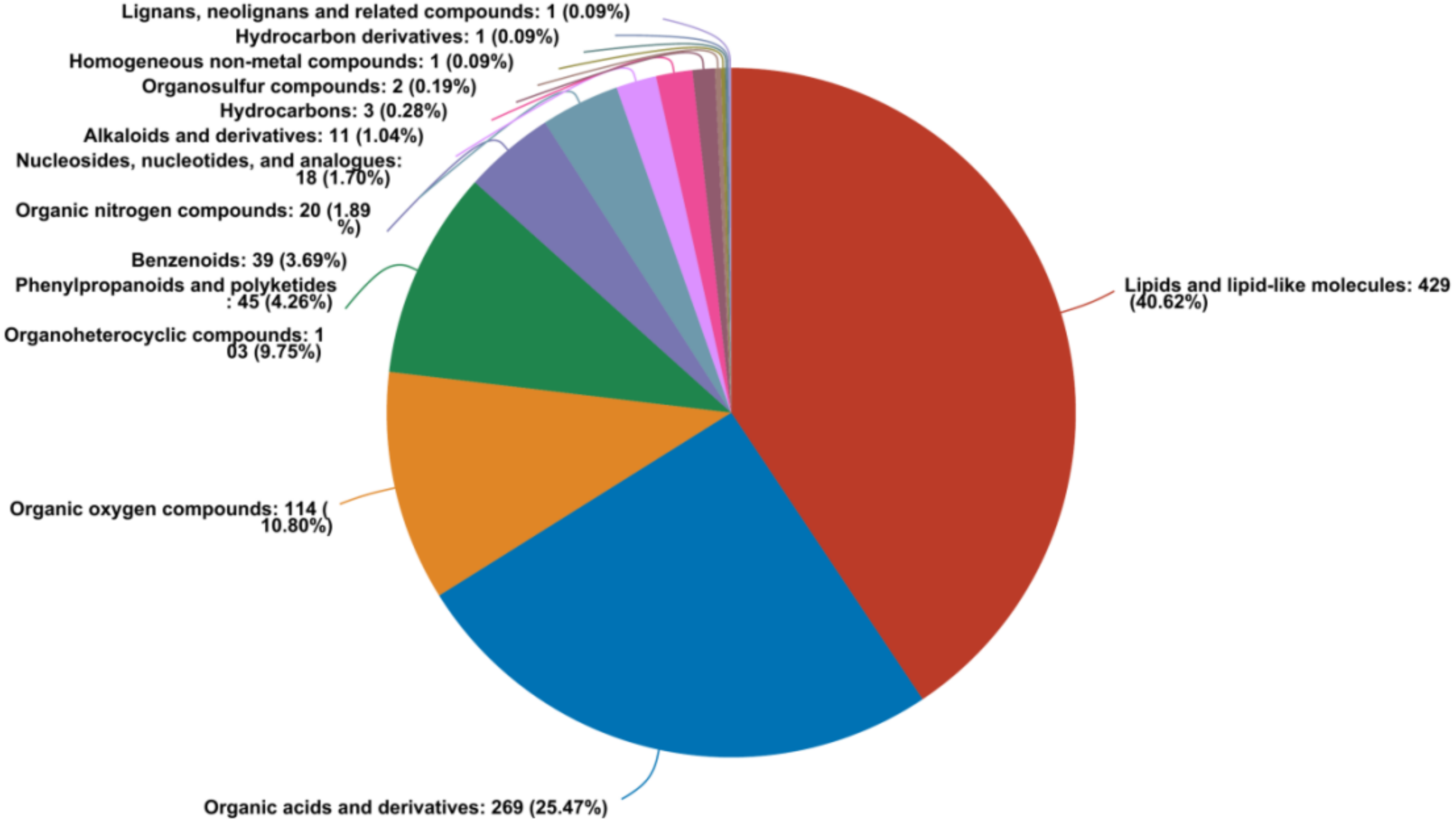

Figure S2 Classification of metabolites
